# Immune Infiltration and Mitochondrial Function in Diabetic Kidney Disease: WGCNA and Machine Learning Identified Hub Genes with Clinical Validation

**DOI:** 10.3390/ijms27114696

**Published:** 2026-05-23

**Authors:** Suyan Duan, Qian Zhou, Ying Shi, Yuyou Ye, Hujia Hua, Dehui Liu, Yuqian Xue, Chengning Zhang, Yanggang Yuan, Changying Xing, Huijuan Mao, Bo Zhang

**Affiliations:** Department of Nephrology, The First Affiliated Hospital with Nanjing Medical University, Nanjing Medical University, 300 Guangzhou Road, Nanjing 210029, China; duansuyan@jsph.org.cn (S.D.); zhouqian77@stu.njmu.edu.cn (Q.Z.); s1240130180@163.com (Y.S.); yyy888@stu.njmu.edu.cn (Y.Y.);

**Keywords:** diabetic kidney disease, bioinformatics analysis, immune infiltration, mitochondria, clinical cohort

## Abstract

Diabetic kidney disease (DKD) lacks specific biomarkers reflecting the interplay between mitochondrial dysfunction and immune microenvironment remodeling. To address this, we integrated multi-dataset transcriptomics (GEO, MitoCarta 3.0, GeneCards) with Weighted Gene Co-expression Network Analysis, protein–protein interaction networks, and machine learning algorithms to identify key diagnostic genes. Single-nucleus RNA sequencing was utilized to map cell-type distributions. Subsequently, a single-center cohort of 70 biopsy-confirmed DKD patients was enrolled for validation of the key hub gene, *HDAC6*. We identified four hub genes: *EGF* (downregulated), *HDAC6*, *TPM1*, and *VCAM1* (upregulated). All genes exhibited robust diagnostic efficacy, and single-nucleus analysis revealed distinct renal cell-type enrichment patterns. Clinically, high renal HDAC6 expression correlated with severe interstitial inflammation, elevated complement C3 and cystatin C, and reduced urinary ammonium (a clinical proxy for proximal tubular mitochondrial dysfunction). Crucially, high HDAC6 served as an independent risk factor for both renal endpoints and cardiorenal composite events. In conclusion, *EGF*, *HDAC6*, *TPM1*, and *VCAM1* are key regulators in DKD. Specifically, intrarenal HDAC6 quantification serves as a precise histological metric for prognostic stratification and underscores its potential as a therapeutic target for DKD intervention.

## 1. Introduction

Diabetic kidney disease (DKD) is one of the most common and serious microvascular complications of diabetes [1,2]. With the rising prevalence of diabetes, DKD has become a leading cause of chronic kidney disease and end-stage kidney disease (ESKD) [3]. Furthermore, patients with DKD are at a higher risk of developing cardiovascular diseases, imposing a heavy health and economic burden on both individuals and society [4]. Current treatment strategies for DKD primarily focus on controlling blood pressure and blood glucose, and inhibiting the renin–angiotensin–aldosterone system (RAAS) [5]. However, due to individual heterogeneity and disease complexity, not all patients achieve satisfactory outcomes [6]. Hence, exploring new specific biomarkers to predict DKD progression, facilitate early intervention, and improve patient prognosis holds significant clinical importance.

The pathogenesis of DKD is complex, and recent studies have emphasized the key roles of mitochondrial dysfunction and immune cell infiltration in the pathophysiology of DKD [7]. Mitochondria are crucial organelles responsible for cellular energy production and metabolic regulation. Under hyperglycemic conditions, elevated blood glucose levels can alter mitochondrial function, leading to excessive generation of reactive oxygen species (ROS). The accumulation of ROS induces oxidative stress, damaging mitochondrial DNA, proteins, and lipids, thereby further exacerbating mitochondrial dysfunction. This damage not only impairs the normal function of renal tubular and glomerular cells, but can also trigger immune responses by releasing damage-associated molecular patterns (DAMPs). DAMPs can be recognized by the innate immune system, recruiting immune cells such as macrophages and T cells to infiltrate renal tissue. Immune cell infiltration initiates and sustains renal inflammatory responses, leading to tubulointerstitial fibrosis and glomerulosclerosis. Mitochondrial dysfunction and immune inflammation are key pathogenic mechanisms in DKD, but the combined action has not been fully elucidated and warrants further investigation.

In this study, we identified diagnostic genes for DKD using a bioinformatic approach combining immune infiltration and mitochondrial dysfunction and validated them.

## 2. Results

### 2.1. Acquisition of Differentially Expressed Mito-Immune-Related Genes (DE-MIRGs)

#### 2.1.1. Identification of Differentially Expressed Mito-Related Genes (DE-MRGs)

**Figure 1:**
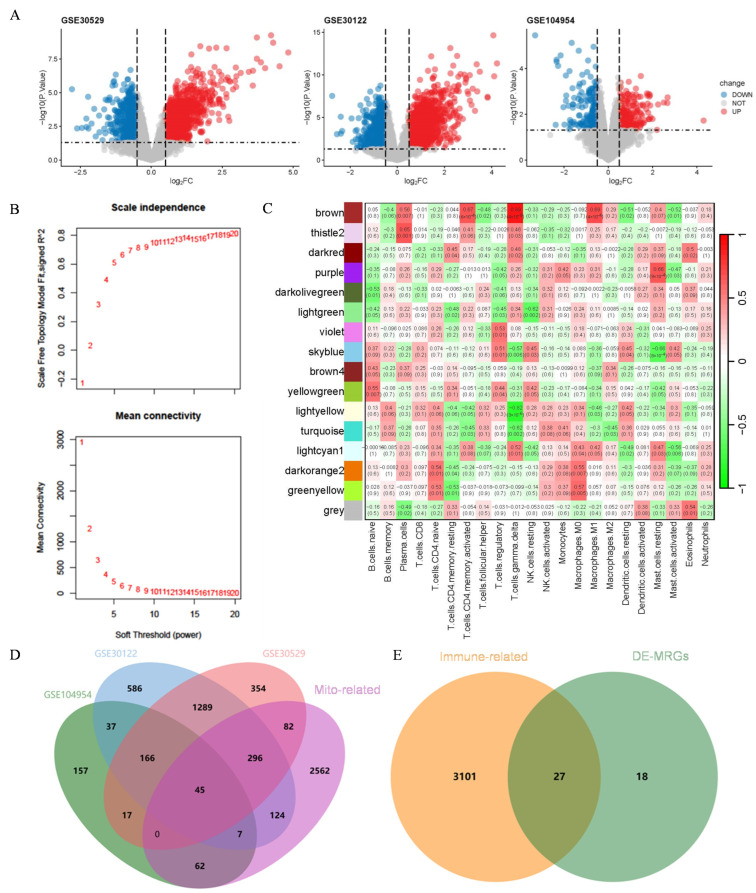
(**A**) Volcano plots of DEGs in the GSE30122, GSE30529, and GSE104954 datasets. (**B**) Selection of the optimal soft-thresholding power of WGCNA. (**C**) Modules identified by WGCNA. (**D**) Venn diagram of DE-MRGs. (**E**) Venn diagram of DE-MIRGs.

A total of 2550, 2249, and 491 Differentially Expressed Genes (DEGs) were identified from the GSE30122, GSE30529, and GSE104954 datasets, respectively (Figure 1A). Among these, 211 genes were differentially expressed across all three datasets. Concurrently, 3178 Mito-related genes were screened from the MitoCarta 3.0 and GeneCards databases. The intersection of these two gene sets yielded 45 DE-MRGs (Figure 1D).

#### 2.1.2. Immune Infiltration Analysis and Identification of Immune-Related Genes

**Figure 2:**
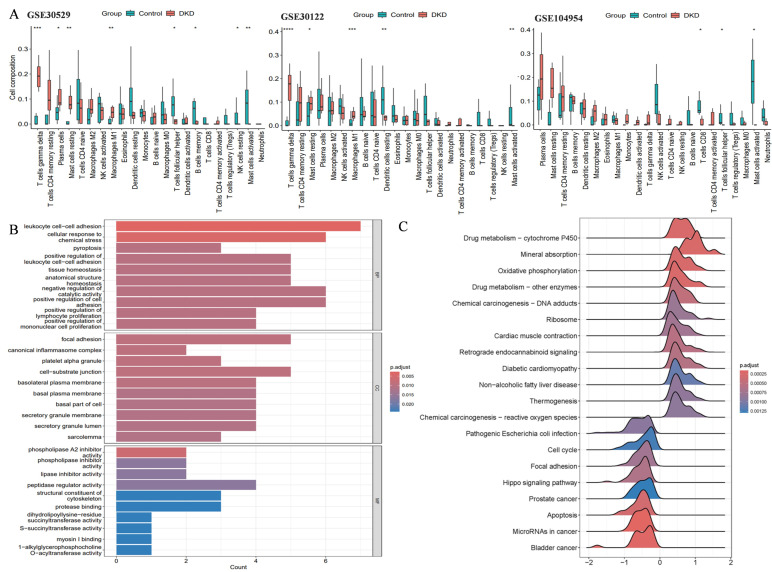
(**A**) Immune cell infiltration profiles in the GSE30529, GSE30122, and GSE104954 datasets (* *p* < 0.05, ** *p* < 0.01, *** *p* < 0.001, **** *p* < 0.0001). (**B**) Bar plot of GO functional enrichment analysis for DE-MIRGs. (**C**) Ridge plot of KEGG enrichment analysis for DE-MIRGs.

Analysis using the CIBERSORT algorithm revealed that in the GSE30529 dataset, eight types of immune cells showed statistically significant differences in infiltration levels in the tubulointerstitial compartment of the DKD group, including plasma cells, CD8-positive T cells (cytotoxic T lymphocytes), regulatory T cells, gamma delta T cells, resting natural killer cells, M1 macrophages, resting mast cells, and activated mast cells (Figure 2A). Meanwhile, in the GSE30122 dataset, five immune cell types showed differential infiltration between the DKD and control groups: regulatory T cells, gamma delta T cells, M1 macrophages, resting dendritic cells, and resting mast cells (Figure 2A). Furthermore, data from GSE104954 indicated distribution differences for three immune cell types: CD8-positive T cells, follicular helper T cells, and resting mast cells (Figure 2A).

For the WGCNA conducted on the GSE30529 dataset, the soft threshold power was set to 16 (scale-free fit index (R^2^) > 0.80, network connectivity < 200) (Figure 1B). A total of 16 co-expression modules were identified (Figure 1C). Among these, the skyblue and brown modules, identified through a systematic screening for modules harboring the maximal count of immune-related genes meeting the stringent threshold (*p* < 0.05 and |r| > 0.4), were selected as Immune-related Genes for subsequent analysis.

#### 2.1.3. Identification and Functional Enrichment Analysis of DE-MIRGs

The intersection of the differentially expressed Mito-related genes with the two key modules mentioned above identified 27 DE-MIRGs (Figure 1E). GO enrichment analysis revealed that in Biological Process (BP), these genes were primarily involved in cell adhesion, cellular response to chemical stimulus, and lymphocyte and monocyte proliferation. In Cellular Component (CC), they were mainly localized to focal adhesion, platelet alpha granule lumen, canonical inflammasome complex, and basolateral/basal plasma membrane. Regarding Molecular Function (MF), key enrichments included phospholipase inhibitor activity, structural constituents of the cytoskeleton, and critically, dihydrolipoamide succinyltransferase activity, a core enzymatic component of the mitochondrial tricarboxylic acid (TCA) cycle (Figure 2B). KEGG pathway analysis indicated that these genes were significantly enriched in pathways associated with energy and metabolic diseases, drug metabolism, and cancer occurrence, among others (Figure 2C).

### 2.2. Screening Hub Genes and Validation

#### 2.2.1. Screening Based on Protein–Protein Interaction (PPI) Network and Machine Learning

**Figure 3:**
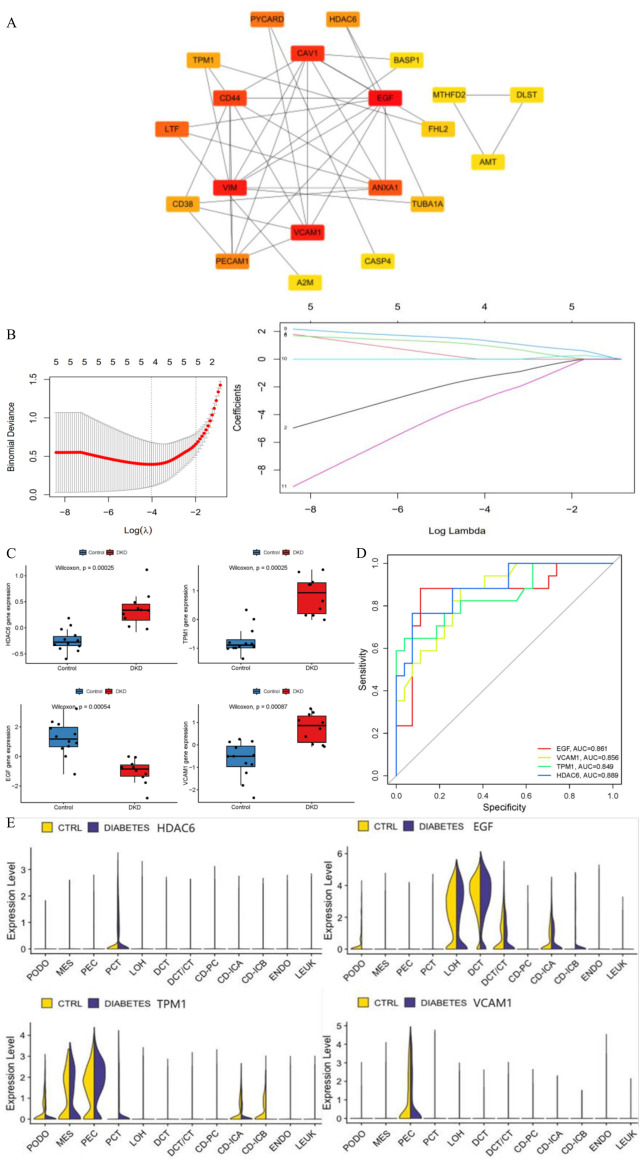
(**A**) PPI network of DE-MIRGs. (**B**) LASSO regression algorithm for candidate genes. The left panel shows the cross-validation error curve, with optimal λ marked by vertical dashed lines; The right panel illustrates coefficient paths, each colored line represents a candidate gene. (**C**) Expression of hub genes in the validation dataset. (**D**) ROC curve analysis of hub genes. (**E**) Distribution of hub genes across 12 cell clusters.

First, based on the PPI network (Figure 3A), 11 candidate genes were screened using the cytoHubba plugin. Then, the LASSO regression algorithm was applied to screen 4 key genes from the 11 genes (Figure 3B). Finally, four hub genes—*EGF*, *HDAC6*, *TPM1*, and *VCAM1*—were identified. Among them, *HDAC6*, *TPM1*, and *VCAM1* were upregulated in DKD renal tissues, while *EGF* was downregulated.

#### 2.2.2. Validation of Hub Gene Expression and Diagnostic Efficacy Evaluation

In the GSE30529 dataset, the expression levels of *HDAC6*, *TPM1*, and *VCAM1* were significantly higher in DKD tissues compared to normal controls, while *EGF* expression was significantly lower in DKD tissues. This expression trend was consistently validated in the validation set, with all differences being statistically significant (Figure 3C). Receiver operating characteristic (ROC) curve analysis was used to evaluate the diagnostic value of the hub genes. In the validation set, the areas under the curve (AUC) for *EGF*, *HDAC6*, *TPM1*, and *VCAM1* were 0.861, 0.889, 0.849, and 0.856, respectively (Figure 3D), indicating their good diagnostic potential.

#### 2.2.3. Cell Type Specific Expression

Through single-nucleus RNA sequencing, we determined the distribution of *EGF*, *HDAC6*, *TPM1*, and *VCAM1* in 12 cell groups (Figure 3E). Specifically, *HDAC6* was specifically highly expressed in proximal tubules; *EGF* showed high expression in podocytes, loops of Henle, distal tubules, and collecting ducts; *TPM1* was mainly localized to podocytes, mesangial cells, parietal epithelial cells, proximal tubules, and collecting ducts; and *VCAM1* was significantly highly expressed in parietal epithelial cells.

#### 2.2.4. Clinical Analysis

Correlation analysis showed that in DKD patients, the expression levels of *HDAC6*, *TPM1*, and *VCAM1* were negatively correlated with estimated glomerular filtration rate (eGFR), positively correlated with serum creatinine (Scr), and negatively correlated with urinary albumin-to-creatinine ratio (UACR). Conversely, *EGF* expression showed a positive correlation trend with eGFR, a negative correlation with Scr, and a positive correlation with UACR levels. All correlations were statistically significant (Table 1).

### 2.3. Semi Quantitative Analysis of HDAC6, Its Association with Clinical Indicators and Its Prognostic Value

Given *HDAC6* exhibited optimal diagnostic performance, tubular-specific expression, and functional relevance to metabolism, inflammation, and autophagy, it was prioritized for proteomic validation in our prospective DKD biopsy cohort.

#### 2.3.1. Immunohistochemistry of HDAC6

**Figure 4:**
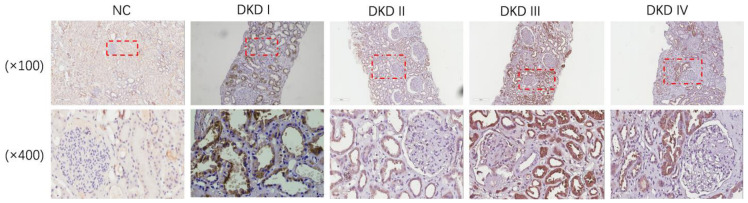
Immunohistochemical images of HDAC6 staining. Representative immunohistochemical images of HDAC6 staining in DKD patients with different classes (magnification ×100 and ×400).

Immunohistochemical results from our center showed that HDAC6 was primarily expressed in renal tubular cells and tubulointerstitial infiltrating cells (Figure 4). Compared to the normal control group, HDAC6 expression levels were significantly increased in the renal tissues of the DKD group.

#### 2.3.2. Characteristics of Enrolled Patients

This study included 70 biopsy-confirmed DKD patients. Based on the tertiles of renal tissue HDAC6 protein expression levels, patients were divided into low-, medium-, and high-expression groups. The median follow-up time for all patients was 32 (14.00, 40.75) months. During follow-up, 40 patients (57.14%) reached the composite kidney endpoint, and 53 patients (75.71%) experienced a major adverse cardiorenal event. Compared to the low-expression group, patients in the high HDAC6 expression group exhibited more severe renal impairment, with significantly higher baseline levels of Scr, cystatin C, and BUN, and significantly lower eGFR levels. Regarding metabolic and biochemical indices, LDL-C and ALP levels were also significantly elevated in the high-expression group. Furthermore, patients in the high-expression group had more severe renal pathological damage, manifested as more advanced pathological stages, a higher proportion of glomerulosclerosis, and more severe interstitial inflammation (IF) scores. Regarding endpoint events, the incidence rates of both the kidney endpoint event (73.91%) and the cardiorenal composite endpoint event (91.30%) were significantly higher in the high HDAC6 expression group compared to the other two groups. Serum complement C3 levels also differed significantly among the three groups. However, baseline characteristics such as age, sex, diabetes duration, blood pressure, HbA1c, and 24hUP showed no statistically significant differences among the three groups (Table S1).

#### 2.3.3. Correlation Between HDAC6 Protein Expression and Immune Microenvironment

To further explore the relationship between HDAC6 expression and the renal immune microenvironment, we analyzed its correlation with renal pathological inflammation and serum immunoglobulin and complement levels. Spearman correlation analysis showed that renal tissue HDAC6 expression level was significantly positively correlated with the IF score and significantly positively correlated with serum complement C3 level (Table 2).

To determine whether HDAC6 expression is an independent factor for aggravated interstitial inflammation, we performed ordinal logistic regression analysis. Univariate analysis showed that high HDAC6 expression was associated with more severe interstitial inflammation. After adjusting for multiple potential confounding factors, high renal tissue HDAC6 expression remained an independent risk factor for an elevated IF score (adjusted OR = 1.22, 95% CI: 1.01–1.48, *p* = 0.049) (Table 3).

#### 2.3.4. Association Between HDAC6 Expression and Tubular Function

To investigate the relationship between renal tissue HDAC6 expression and tubular function, we performed Spearman correlation analysis. The results showed that among several tubular injury and metabolic indicators, HDAC6 expression level was significantly negatively correlated with urinary ammonium excretion and significantly positively correlated with cystatin C level. Specifically, the correlation coefficient between HDAC6 expression and urinary ammonium was r = −0.363, *p* = 0.042, and with cystatin C, it was r = 0.291, *p* = 0.014 (Table 4).

#### 2.3.5. Relationship Between HDAC6 Expression and Cardiorenal Prognosis

**Figure 5:**
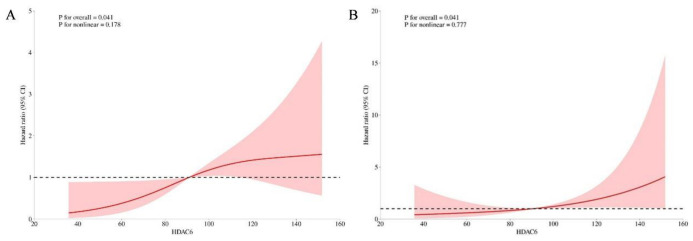
Dose–response relationship between HDAC6 expression level and the risk of poor prognosis in DKD patients. Dashed lines indicate the null value (hazard ratio = 1.0). (**A**) Composite kidney outcome. (**B**) Major adverse cardiorenal event outcome adjusting for sex, age, BMI, cystatin C, BUN, albumin, and eGFR.

We used a restricted cubic spline (RCS) model to analyze the dose–response relationship between HDAC6 expression level and endpoint events. The results showed a significant linear association between HDAC6 expression level and the risk of the composite kidney endpoint (*p* for overall = 0.041, *p* for nonlinear = 0.178) (Figure 5A). Similarly, a significant linear dose–response relationship was also observed with the risk of major adverse cardiorenal events (*p* for overall = 0.041, *p* for nonlinear = 0.777) (Figure 5B). The predictive value of HDAC6 expression for prognosis was evaluated using Cox proportional hazards regression models. In the unadjusted model, the high HDAC6 expression group (median split) was associated with a significantly increased risk of both the composite kidney endpoint and the cardiorenal composite endpoint. After adjusting for potential confounders, high HDAC6 expression remained an independent risk factor for the composite kidney endpoint event (HR: 2.08 [1.02–4.26], *p* = 0.045). Similarly, in the multivariate analysis for the cardiorenal composite endpoint, after adjusting for potential confounders, high HDAC6 expression was also independently associated with a significantly increased risk of the cardiorenal composite endpoint (HR: 1.87 [1.05–3.33], *p* = 0.034) (Table 5).

## 3. Discussion

DKD is a leading cause of ESKD. Its pathogenesis is complex, involving the interaction of multiple factors such as metabolic disorders, hemodynamic alterations, inflammatory responses, and fibrosis [2]. In recent years, increasing research has emphasized the critical roles of renal immune microenvironment dysregulation and cellular metabolic dysfunction, particularly mitochondrial dysfunction, in the onset and progression of DKD [8,9]. Therefore, it is necessary to conduct further research to identify potential biomarkers and therapeutic targets. Through integrated bioinformatics analysis, this study focused on the “immune infiltration–mitochondrial function” axis and identified four hub genes (*EGF*, *HDAC6*, *TPM1*, *VCAM1*), and all hub genes had excellent diagnostic efficacy. Given that *HDAC6* demonstrated the best diagnostic performance, exhibited characteristic expression at the tubular level in single-cell analysis, we verified its association with clinical indicators and prognostic value in our center.

EGF is an important mitogenic peptide that plays a key role in kidney development, repair, and maintaining the homeostasis of renal tubular epithelial cells [10]. This study corroborates that renal EGF downregulation signifies the depletion of intrinsic repair capacity in DKD [11,12]. Aligning with Keller et al. and Geurts et al. [13,14], we found reduced EGF correlated with rapid progression and diminished distal nephron mass. Single-cell data localized EGF to podocytes and distal tubules, reinforcing its role as a marker of epithelial differentiation [15]. Intriguingly, recent evidence suggests EGF deficiency may exacerbate mitochondrial dysfunction and necroptosis in injured tubules, thereby amplifying local sterile inflammation [16]. While exogenous EGF shows renoprotective potential [17], the upstream mechanisms of its suppression in humans require further clarification.

VCAM1 is a member of the immunoglobulin superfamily, primarily expressed on activated endothelial cells. VCAM1 was confirmed as a key mediator of microinflammation, with its expression correlating with declining renal function [18,19]. Notably, our single-cell analysis revealed predominant expression in parietal epithelial cells, differing from studies emphasizing tubular expression in advanced disease [20]. This aligns with the concept that VCAM1 sources shift dynamically: from early parietal epithelial stress to a later association with failed tubular repair [21,22]. Critically, sustained VCAM1 upregulation driven by NF-κB signaling not only recruits inflammatory cells but also imposes a significant metabolic burden on resident renal cells, exacerbating mitochondrial respiratory dysfunction and reactive oxygen species (ROS) overproduction [9].

HDAC6 is a Class IIb histone deacetylase, emerging as a central hub linking metabolic dysfunction to immune dysregulation. Beyond regulating inflammation and fibrosis [23,24,25], recent biochemical evidence confirms HDAC6 directly alters fumarate hydratase activity and mitochondrial cristae structure [26]. In our cohort, HDAC6 was upregulated in proximal tubules and independently predicted adverse cardiorenal outcomes. Crucially, its expression correlated with reduced urinary ammonium excretion [27], a clinical proxy for proximal tubular mitochondrial energy dysfunction, and elevated interstitial inflammation. Mechanistically, HDAC6 exacerbates mitochondrial damage by promoting Drp1-dependent mitochondrial fission and NLRP3 inflammasome activation [28]. While pharmacological inhibition alleviates DKD in models [25], its exact therapeutic efficacy requires prospective validation [29].

TPM1, a cytoskeletal stabilizer, was upregulated in DKD tissues. Given the cytoskeleton governs mitochondrial dynamics and transport [30], aberrant TPM1 likely disrupts mitochondrial distribution and morphology, thereby exacerbating metabolic stress and fibrosis [31]. Specifically, TPM1-mediated cytoskeletal remodeling can impair the interaction between mitochondria and the endoplasmic reticulum, leading to defective mitochondrial quality control and subsequent inflammatory activation [31]. Current evidence for TPM1 in DKD remains nascent, warranting deeper exploration into its specific coupling with mitochondrial dysfunction.

Diabetic kidney disease involves complex interactions between mitochondrial dysfunction and immune dysregulation, amplified by chronic hyperglycemia. Our study employed a multi-stage strategy to elucidate this pathogenesis. Integrating multi-cohort transcriptomics with WGCNA, we identified 27 genes intersecting mitochondrial function and immune regulation. Functional enrichment mapped these genes to the TCA cycle, inflammasome complexes, and cytoskeletal dynamics. Machine learning refined this signature to four diagnostic hub genes: *EGF*, *HDAC6*, *TPM1*, and *VCAM1*. We validated their expression across independent datasets and resolved their specific renal cell types using single-nucleus RNA sequencing. Crucially, our prospective clinical cohort bridged molecular findings to pathophysiology. Renal HDAC6 expression correlated with reduced urinary ammonium, a marker of tubular mitochondrial dysfunction, and interstitial inflammation, while independently predicting adverse cardiorenal outcomes. This systematic integration of bioinformatics screening with histopathological validation provides a robust framework for identifying biomarkers linking mitochondrial dysfunction to immune dysregulation in DKD.

However, this study has several limitations. First, the core findings originated from bioinformatics mining of public transcriptomic data with a limited training sample size. Although supplemented with clinicopathological correlation analysis in an independent cohort, direct evidence linking these genes to mitochondrial function and specific local immune microenvironment remodeling remains lacking. Second, the Mito-related genes used to construct the network in this study have a broad scope. The screened hub genes may belong to mitochondrial function-related regulatory networks rather than being mitochondrial resident proteins. Third, immune infiltration deconvolution results based on algorithms like CIBERSORT are susceptible to variations in parenchymal cell proportions and should be interpreted as trend references. Finally, protein-level validation was limited to HDAC6. The cell-specific protein expression, post-translational modifications, and functional significance of EGF, TPM1, and VCAM1 in human DKD tissues remain unvalidated.

## 4. Materials and Methods

### 4.1. Data Sources

This study analyzed three DKD datasets: GSE30529 (platform GPL571) comprising 10 DKD samples and 12 control samples; GSE30122 (platform GPL571) comprising 10 DKD samples and 24 control samples; and GSE104954 (platform GPL22945) comprising 7 DKD samples and 3 control samples. GSE30529 served as the training set. The GSE30122 and GSE104954 datasets were combined to form validation set (Figure S1). A total of 1136 genes localized to mitochondria were obtained from the human MitoCarta 3.0 database (https://www.broadinstitute.org/mitocarta/mitocarta30-inventory-mammalian-mitochondrial-proteins-and-pathways, accessed on 1 May 2024), and 2961 genes were extracted from the GeneCards database. After merging and removing duplicates, 3178 Mito-related genes were obtained.

### 4.2. Identification of Differentially Expressed Genes

Using the criteria of |log2 Fold Change (FC)| > 0.5 and *p* < 0.05, the limma (v3.60.0) R packagewas utilized to perform differential expression analysis on the GSE30122, GSE30529, and GSE104954 datasets. Genes with log2FC > 0.5 and *p* < 0.05 were defined as upregulated genes, while those with log2FC < −0.5 and *p* < 0.05 were defined as downregulated genes. The heatmap and volcano plot of DEGs were generated using the the pheatmap (v1.0.12) and ggplot2 (v3.5.1) R packages, respectively. Subsequently, the resulting DEGs were intersected with the 3178 Mito-related genes to obtain DE-MRGs.

### 4.3. Immune Infiltration Analysis and WGCNA

The CIBERSORT algorithm estimates the composition and abundance of 22 immune cell types within mixed cell populations based on transcriptomic data via a deconvolution algorithm. This study first employed this algorithm to assess the proportions of immune cells in normal and DKD samples from the GSE30529 dataset [32]. WGCNA was used to identify modules of highly correlated genes and to analyze the associations between modules and with immune cell infiltration characteristics, aiming to discover candidate biomarkers or therapeutic targets. The R package WGCNA (v1.72-5) was used to construct the co-expression network [33], and identify modules most associated with immune cells in DKD patients. Specific steps included: data preprocessing and removal of outlier samples; construction of a correlation matrix and selection of the optimal soft threshold for converting it into an adjacency matrix; generation of a topological overlap matrix based on the adjacency matrix; and using dissimilarity measures based on the topological overlap matrix to cluster genes with similar expression patterns into modules via average linkage hierarchical clustering. Finally, the two modules with the strongest correlation to immune cells were selected as key modules for subsequent analysis. The intersection of DEMGs and the genes within the key modules yielded genes defined as DE-MIRGs for further investigation.

### 4.4. GO and KEGG Functional Enrichment Analysis

Gene Ontology (GO) and Kyoto Encyclopedia of Genes and Genomes (KEGG) pathway enrichment analyses were performed on the DE-MIRGs gene set using the R package clusterProfiler (v4.10.1) to assess their associated biological processes, molecular functions, cellular components, and signaling pathways.

### 4.5. Screening Hub Genes Based on Protein–Protein Interaction Network and Machine Learning

The STRING database was used to construct a Protein–Protein Interaction (PPI) network for the DE-MIRGs, and Cytoscape (v3.10.3) software was utilized for visualization. Genes in the network were scored using nine algorithms (EcCentricity, MCC, MNC, Radiality, Stress, Betweenness, EPC, Closeness, and Degree) from Cytoscape’s cytoHubba plugin. The top 50% of genes from each algorithm were taken, and their intersection was considered as a set of candidate hub genes [34]. Subsequently, Least Absolute Shrinkage and Selection Operator (LASSO) logistic regression analysis was employed to screen for hub genes from the identified candidates [35].

### 4.6. Clinical Correlation Analysis

The Nephroseq v5 database (https://nephroseq.org/, accessed on 1 May 2024) is a comprehensive platform for evaluating correlations between gene expression levels and clinical features of kidney diseases [36]. This study utilized this database to explore the correlations between hub gene expression and clinical characteristics of DKD.

### 4.7. Single-Nucleus RNA Sequencing Analysis

The Kidney Integrative Transcriptomics (K.I.T.) database, developed by Dr. Ben Humphrey’s laboratory at the University of Washington, is a single-cell sequencing database for kidney diseases. This study utilized this database to analyze the expression distribution of hub genes across different kidney cell types and visualized the results.

Given that *HDAC6* demonstrated the best diagnostic performance in validation data, exhibited characteristic expression at the tubular level in single-cell analysis, and is closely associated with pathways related to cell metabolism, inflammation, and autophagy as a deacetylase, aligning closely with the theme of “immune infiltration–mitochondrial function” in this study, it was selected as the representative gene for in-depth validation and exploration of mechanistic associations at the tissue protein level in a prospectively collected DKD biopsy cohort from our center.

### 4.8. Study Subjects

Seventy patients with biopsy-confirmed DKD diagnosed at the First Affiliated Hospital of Nanjing Medical University between January 2017 and May 2021 were selected. Exclusion criteria: ① age < 18 years; ② patients with acute kidney injury; ③ patients with <5 glomeruli obtained by biopsy; ④ patients with other severe systemic diseases or other glomerular diseases. Five samples of adjacent normal tissues were collected 0.5 cm from the tumor margin and pathologically confirmed as normal tissue. This study was approved by the Ethics Committee of the First Affiliated Hospital of Nanjing Medical University (Approval No.: 2021-SR-398), and all participants provided informed consent.

### 4.9. Clinical Data

The following clinical data were collected at the time of renal biopsy: age, sex, blood pressure, fasting plasma glucose (FPG), hemoglobin A1c (HbA1c), blood urea nitrogen (BUN), serum creatinine (Scr), 24-h urine protein (24hUP), high-density lipoprotein cholesterol (HDL-C), low-density lipoprotein cholesterol (LDL-C), parathyroid hormone (PTH), retinol-binding protein (RBP), alkaline phosphatase (ALP), 25-hydroxyvitamin D (25(OH)D), urinary N-acetyl-β-D-glucosaminidase (NAG), serum/urine neutrophil gelatinase-associated lipocalin (NGAL), and medication use. Kidney specimens were routinely examined by two experienced pathologists using light microscopy, immunofluorescence, and electron microscopy, with at least 5 glomeruli obtained from each biopsy specimen. DKD pathological staging was performed based on HE staining, PAS staining, silver staining, Masson trichrome staining under light microscopy, combined with electron microscopy findings. Diagnostic criteria were as follows [37]: Class I, no or minimal lesions by light microscopy, with glomerular basement membrane thickening by electron microscopy (thickness >430 nm for males ≥9 years, >395 nm for females ≥9 years); Class IIa, mild mesangial expansion (≤25% of mesangial area); Class IIb, severe mesangial expansion (>25% of mesang area); Class III, presence of Kimmelstiel–Wilson nodules; Class IV, advanced diabetic glomerulosclerosis (>50% global glomerulosclerosis). Interstitial fibrosis and tubular atrophy (IFTA) and interstitial inflammation (IF) were semi-quantitatively scored based on the proportion of the cortical area involved (Score 0: absent; 1: <25%; 2: 25–50%; 3: >50%). Vascular lesions were scored based on the presence of arteriolar hyalinosis and large vessel arteriosclerosis. Two expert pathologists reviewed and resolved any scoring discrepancies until consensus was reached.

### 4.10. Immunohistochemistry

Paraffin-embedded tissue sections (2 μm) were dewaxed, subjected to antigen retrieval in EDTA buffer for 20 min, and washed with phosphate-buffered saline (PBS). Sections were blocked with 5% bovine serum albumin at room temperature for 1 h, then incubated with HDAC6 antibody (1:200, Cell Signaling Technology, CST, Danvers, MA, USA) overnight at 4 °C. After washing with PBS, sections were incubated with secondary antibody at 37 °C for 1 h. Sections were then stained with DAB for 40 s, counterstained with hematoxylin, and finally dehydrated and mounted.

For each section, 8–10 random fields were captured under a microscope. Semi-quantitative analysis of immunohistochemical images was performed using ImageJ (v1.54j) software. Images were first converted to grayscale, thresholds were set to identify positively DAB-stained areas, and their integrated optical density (IOD) and corresponding area were extracted. The mean optical density (MOD) was calculated using the formula IOD/Area to reflect the expression level of HDAC6.

### 4.11. Follow-Up and Observation Endpoints

All patients were followed up via telephone or outpatient system from the time of renal biopsy until the occurrence of a composite kidney endpoint or major adverse cardiovascular events. The primary endpoint was a composite kidney endpoint, defined as the occurrence of any of the following events: (1) a sustained doubling of the Scr level from the baseline value (defined as the measurement at the time of biopsy hospitalization); (2) progression to ESKD. The key secondary endpoint was a major adverse cardiorenal event, defined as the occurrence of any of the following events: (1) the aforementioned “composite kidney endpoint”; (2) a major adverse cardiovascular event, including coronary events (e.g., myocardial infarction, revascularization), cerebrovascular events (e.g., stroke), peripheral vascular events, and death due to myocardial infarction or stroke [38]. Follow-up ended on 1 January 2024.

### 4.12. Statistical Analysis

Statistical analyses were performed using R software (v4.2.1). Receiver operating characteristic (ROC) curve analysis was used to evaluate the diagnostic value of hub genes. Pearson correlation analysis was used to assess the correlation between hub gene expression and clinical features. Unpaired *t*-test was used to evaluate the expression differences in hub genes between DKD and control samples. Data are presented as mean ± standard deviation (SD), median with interquartile range, or percentage. Comparisons between groups were performed using one-way analysis of variance (ANOVA), Kruskal–Wallis test, or chi-square test (χ^2^), as appropriate based on data distribution and study context. Correlations between HDAC6 expression and clinical data were analyzed using Pearson or Spearman tests. Cox proportional hazards models and restricted cubic splines (RCS) were used to assess the relationship between HDAC6 expression and cardiorenal outcomes. *p* < 0.05 was considered statistically significant.

## 5. Conclusions

This study established an integrated screening strategy combining multi-omics mining with machine learning, identifying *EGF*, *HDAC6*, *TPM1*, and *VCAM1* as key diagnostic candidates for diabetic kidney disease. Clinically, we established renal HDAC6 as an independent prognostic biomarker linking immune-inflammatory dysregulation to adverse cardiorenal outcomes. Collectively, this study provides preliminary computational and clinical evidence for this molecular network and highlights intrarenal HDAC6 quantification as a promising histological metric for risk stratification and therapeutic targeting.

## Figures and Tables

**Table 1:** Correlation analysis of hub genes with eGFR, Scr, and UACR.

Hub Genes	Indicator	Sample Size (*n*)	*p*	r	R^2^
*HDAC6*	eGFR	186	<0.001	−0.622	0.386
	Scr	17	0.023	0.548	0.300
	UACR	17	0.029	0.804	0.648
*EGF*	eGFR	186	<0.001	0.665	0.443
	Scr	17	0.005	−0.646	0.417
	UACR	17	0.037	−0.751	0.564
*TPM1*	eGFR	186	<0.001	−0.635	0.403
	Scr	17	0.032	0.520	0.271
	UACR	17	0.046	0.595	0.354
*VCAM1*	eGFR	186	<0.001	−0.524	0.274
	Scr	17	0.013	0.649	0.421
	UACR	17	0.027	0.265	0.070

**Table 2:** Correlation between HDAC6 and immune markers.

	R	*p*
IF (0/1/2)	0.258	0.031
IgA (g/L)	0.027	0.746
IgG (g/L)	−0.089	0.278
C3 (g/L)	0.201	0.001
C4 (g/L)	0.093	0.256

**Table 3:** Logistic regression analysis of the association between HDAC6 expression and interstitial inflammation score.

	Univariate			Multivariate		
	β	*p*	OR (95%CI)	β	*p*	OR (95%CI)
HDAC6	0.17	0.039	1.19 (1.01~1.40)	0.20	0.049	1.22 (1.01~1.48)

Multivariate model adjusting for sex, age, BMI, eGFR, 24-h urine protein, and pathological stage.

**Table 4:** Correlation between HDAC6 and tubular function indices.

	R	*p*
RBP (mg/L)	0.164	0.175
Urine NAG (U/L)	0.039	0.747
Serum NGAL (ng/mL)	0.095	0.266
Urine NGAL (ng/mL)	0.080	0.377
Urine-specific gravity	0.152	0.069
Urine pH (<5/5–8/>8)	0.066	0.486
Urine bicarbonate (mmol/L)	0.059	0.483
Urine titratable acid (mmol/L)	0.016	0.857
Urine ammonium (mmol/L)	−0.363	0.042
Cystatin C (mg/L)	0.291	0.014
IFTA (0/1/2/3)	0.102	0.398

**Table 5:** Cox proportional hazards regression analysis for the association between HDAC6 expression level and cardiorenal prognosis in DKD patients.

	Univariate			Multivariate		
	β	*p*	HR (95%CI)	β	*p*	HR (95%CI)
Renal Composite Outcome	0.72	0.030	2.05 (1.07~3.93)	0.73	0.045	2.08 (1.02~4.26)
Major Cardiorenal Event	0.59	0.041	1.80 (1.02~3.15)	0.62	0.034	1.87 (1.05~3.33)

Multivariate model adjusting for sex, age, BMI, eGFR, cystatin C, BUN, and 24-h urine protein.

## Data Availability

The transcriptomic datasets (GSE30529, GSE30122, GSE104954) analyzed in the current study are publicly available in the Gene Expression Omnibus (GEO) repository (https://www.ncbi.nlm.nih.gov/geo/, accessed on 1 May 2024). The Mito-related gene list is available from the MitoCarta 3.0 database (https://www.broadinstitute.org/mitocarta/mitocarta30-inventory-mammalian-mitochondrial-proteins-and-pathways, accessed on 1 May 2024) and GeneCards (https://www.GeneCards.org/, accessed on 1 May 2024). The single-nucleus RNA sequencing data were analyzed via the Kidney Integrative Transcriptomics (K.I.T.) database (https://humphreyslab.com/SingleCell/, accessed on 1 May 2024). The clinical correlation data were analyzed using the Nephroseq v5 platform (https://nephroseq.org/, accessed on 1 May 2024). The original clinical data generated and analyzed during the prospective cohort study are not publicly available due to patient privacy and confidentiality restrictions but are available from the corresponding author upon reasonable request.

## Supplementary Materials

The following supporting information can be downloaded at: https://www.mdpi.com/article/10.3390/ijms27114696/s1.

## Abbreviations

AUC	Area Under the Curve
ANOVA	Analysis of Variance
BMI	Body Mass Index
BUN	Blood Urea Nitrogen
CI	Confidence Interval
CST	Cell Signaling Technology
DAMPs	Damage-Associated Molecular Patterns
DBP	Diastolic Blood Pressure
DEGs	Differentially Expressed Genes
DE-MIRGs	Differentially Expressed Immune-related Mitochondria Genes
DEMGs	Differentially Expressed Mitochondria-related Genes
DKD	Diabetic Kidney Disease
DM	Diabetes Mellitus
eGFR	Estimated Glomerular Filtration Rate
EGF	Epidermal Growth Factor
ESKD	End-Stage Kidney Disease
FC	Fold Change
FPG	Fasting Plasma Glucose
GEO	Gene Expression Omnibus
GO	Gene Ontology
Hb	Hemoglobin
HbA1c	Hemoglobin A1c
HDAC6	Histone Deacetylase 6
HDL-C	High-Density Lipoprotein Cholesterol
HR	Hazard Ratio
IF	Interstitial Inflammation
IFTA	Interstitial Fibrosis and Tubular Atrophy
IgA	Immunoglobulin A
IgG	Immunoglobulin G
IOD	Integrated Optical Density
KEGG	Kyoto Encyclopedia of Genes and Genomes
K.I.T.	Kidney Integrative Transcriptomics
LASSO	Least Absolute Shrinkage and Selection Operator
LDL-C	Low-Density Lipoprotein Cholesterol
MOD	Mean Optical Density
NAG	N-acetyl-β-D-glucosaminidase
NGAL	Neutrophil Gelatinase-Associated Lipocalin
OR	Odds Ratio
PBS	Phosphate-Buffered Saline
PPI	Protein–Protein Interaction
PTH	Parathyroid Hormone
RAAS	Renin–Angiotensin–Aldosterone System
RBP	Retinol-Binding Protein
RCS	Restricted Cubic Spline
R^2^	Coefficient of Determination
ROC	Receiver Operating Characteristic
ROS	Reactive Oxygen Species
r	Correlation Coefficient
SBP	Systolic Blood Pressure
Scr	Serum Creatinine
SD	Standard Deviation
TC	Total Cholesterol
TG	Triglyceride
TGF-β	Transforming Growth Factor-β
TPM1	Tropomyosin 1
UACR	Urinary Albumin-to-Creatinine Ratio
VCAM1	Vascular Cell Adhesion Molecule 1
WGCNA	Weighted Gene Co-expression Network Analysis
24hUP	24-h Urine Protein
25(OH)D	25-hydroxyvitamin D

## References

[1] Bloomgarden Z.T. (2005). Diabetic nephropathy. Diabetes Care.

[2] Alicic R.Z., Rooney M.T., Tuttle K.R. (2017). Diabetic Kidney Disease: Challenges, Progress, and Possibilities. Clin. J. Am. Soc. Nephrol..

[3] Barutta F., Bellini S., Canepa S., Durazzo M., Gruden G. (2022). Correction to: Novel biomarkers of diabetic kidney disease: Current status and potential clinical application. Acta Diabetol..

[4] Scilletta S., Di Marco M., Miano N., Filippello A., Di Mauro S., Scamporrino A., Musmeci M., Coppolino G., Di Giacomo Barbagallo F., Bosco G. (2023). Update on Diabetic Kidney Disease (DKD): Focus on Non-Albuminuric DKD and Cardiovascular Risk. Biomolecules.

[5] Palmer S.C., Tendal B., Mustafa R.A., Vandvik P.O., Li S., Hao Q., Tunnicliffe D., Ruospo M., Natale P., Saglimbene V. (2022). Sodium-glucose cotransporter protein-2 (SGLT-2) inhibitors and glucagon-like peptide-1 (GLP-1) receptor agonists for type 2 diabetes: Systematic review and network meta-analysis of randomised controlled trials. BMJ.

[6] Tuomi T., Santoro N., Caprio S., Cai M., Weng J., Groop L. (2014). The many faces of diabetes: A disease with increasing heterogeneity. Lancet.

[7] Saxena S., Mathur A., Kakkar P. (2019). Critical role of mitochondrial dysfunction and impaired mitophagy in diabetic nephropathy. J. Cell. Physiol..

[8] Tang S.C.W., Yiu W.H. (2020). Innate immunity in diabetic kidney disease. Nat. Rev. Nephrol..

[9] Zhang T., Wu J., Zhang J., Hu Y., Zhao Y., Mao G., Jiao J., Wang J., Chen R., Zheng C. (2025). Mitochondrial Dysfunction and Immune Cell Infiltration in Diabetic Kidney Disease: A Mendelian Randomization and Multiomics Study. Mediat. Inflamm..

[10] Hussain M.M. (2001). Structural, biochemical and signaling properties of the low-density lipoprotein receptor gene family. Front. Biosci..

[11] Harris R.C., Zhang M.Z. (2026). The Role of the Epidermal Growth Factor Receptor in Kidney Tubulointerstitial Fibrosis. Semin. Nephrol..

[12] Lautrette A., Li S., Alili R., Sunnarborg S.W., Burtin M., Lee D.C., Friedlander G., Terzi F. (2005). Angiotensin II and EGF receptor cross-talk in chronic kidney diseases: A new therapeutic approach. Nat. Med..

[13] Keller F., Denicolo S., Leierer J., Kruus M., Heinzel A., Kammer M., Ju W., Nair V., Burdet F., Ibberson M. (2024). Association of Urinary Epidermal Growth Factor, Fatty Acid-Binding Protein 3, and Vascular Cell Adhesion Molecule 1 Levels with the Progression of Early Diabetic Kidney Disease. Kidney Blood Press. Res..

[14] Geurts F., van Heugten M.H., Blijdorp C.J., Fenton R.A., Chaker L., Hoorn E.J. (2025). Urinary EGF Reflects Distal Tubular Mass and Is Associated with Hypertension, Serum Magnesium, and Kidney Outcomes. Kidney360.

[15] Harris R.C. (2021). The epidermal growth factor receptor axis and kidney fibrosis. Curr. Opin. Nephrol. Hypertens..

[16] Wang Y., Zhang L., Peng Z. (2023). Investigating EGF and PAG1 as necroptosis-related biomarkers for diabetic nephropathy: An in silico and in vitro validation study. Aging.

[17] Chen J., Chen J.K., Nagai K., Plieth D., Tan M., Lee T.C., Threadgill D.W., Neilson E.G., Harris R.C. (2012). EGFR signaling promotes TGFβ-dependent renal fibrosis. J. Am. Soc. Nephrol..

[18] Cook-Mills J.M., Johnson J.D., Deem T.L., Ochi A., Wang L., Zheng Y. (2004). Calcium mobilization and Rac1 activation are required for VCAM-1 (vascular cell adhesion molecule-1) stimulation of NADPH oxidase activity. Biochem. J..

[19] Tomita-Yagi A., Ozeki-Okuno N., Watanabe-Uehara N., Komaki K., Umehara M., Sawada-Yamauchi H., Minamida A., Sunahara Y., Matoba Y., Nakamura I. (2024). The importance of proinflammatory failed-repair tubular epithelia as a predictor of diabetic kidney disease progression. iScience.

[20] Melchinger I., Guo K., Li X., Guo J., Cantley L.G., Xu L. (2024). VCAM-1 mediates proximal tubule-immune cell cross talk in failed tubule recovery during AKI-to-CKD transition. Am. J. Physiol. Ren. Physiol..

[21] You J., Wang Z., Xu S., Zhang W., Fang Q., Liu H., Peng L., Deng T., Lou J. (2016). Advanced Glycation End Products Impair Glucose-Stimulated Insulin Secretion of a Pancreatic β-Cell Line INS-1-3 by Disturbance of Microtubule Cytoskeleton via p38/MAPK Activation. J. Diabetes Res..

[22] Deng Y., Zhang S., Luo Z., He P., Ma X., Ma Y., Wang J., Zheng L., Tian N., Dong S. (2024). VCAM1: An effective diagnostic marker related to immune cell infiltration in diabetic nephropathy. Front. Endocrinol..

[23] Qu F., Zhao Q., Jin Y. (2025). Histone deacetylase 6: A new player in oxidative stress-associated disorders and cancers (Review). Int. J. Mol. Med..

[24] Zheng Y., Zhang T.N., Hao P.H., Yang N., Du Y. (2025). Histone deacetylases and their inhibitors in kidney diseases. Mol. Ther..

[25] Hou Q., Kan S., Wang Z., Shi J., Zeng C., Yang D., Jiang S., Liu Z. (2022). Inhibition of HDAC6 With CAY10603 Ameliorates Diabetic Kidney Disease by Suppressing NLRP3 Inflammasome. Front. Pharmacol..

[26] Roe A., Dowling C.M., D’Arcy C., Alencar Rodrigues D., Wang Y., Hiller M., Keogh C., Hollinshead K.E.R., Garre M., Cavanagh B. (2025). Inhibition of HDAC6 alters fumarate hydratase activity and mitochondrial structure. Nat. Commun..

[27] Kang H.M., Ahn S.H., Choi P., Ko Y.A., Han S.H., Chinga F., Park A.S., Tao J., Sharma K., Pullman J. (2015). Defective fatty acid oxidation in renal tubular epithelial cells has a key role in kidney fibrosis development. Nat. Med..

[28] Ke B., Chen Y., Tu W., Ye T., Fang X., Yang L. (2018). Inhibition of HDAC6 activity in kidney diseases: A new perspective. Mol. Med..

[29] Zhang Q.Q., Zhang W.J., Chang S. (2023). HDAC6 inhibition: A significant potential regulator and therapeutic option to translate into clinical practice in renal transplantation. Front. Immunol..

[30] Gunning P.W., Schevzov G., Kee A.J., Hardeman E.C. (2005). Tropomyosin isoforms: Divining rods for actin cytoskeleton function. Trends Cell Biol..

[31] Wu C.L., Lee G.H., Chen B.Y., Kuo C.H., Chan T.T., Wang Y.K., Lin S.L., Wong T.Y., Tang M.J. (2025). Depletion of tropomyosin 1.6 alleviates TGF-β1-induced fibroblast activation by promoting the α-SMA-MMP9 matrix-degrading structure. iScience.

[32] Sui S., An X., Xu C., Li Z., Hua Y., Huang G., Sui S., Long Q., Sui Y., Xiong Y. (2020). An immune cell infiltration-based immune score model predicts prognosis and chemotherapy effects in breast cancer. Theranostics.

[33] Zhang B., Horvath S. (2005). A general framework for weighted gene co-expression network analysis. Stat. Appl. Genet. Mol. Biol..

[34] Qu L.H., Luo W.J., Yan Z.G., Liu W.P. (2022). FAM171B as a Novel Biomarker Mediates Tissue Immune Microenvironment in Pulmonary Arterial Hypertension. Mediat. Inflamm..

[35] Zhang J., Yu R., Guo X., Zou Y., Chen S., Zhou K., Chen Y., Li Y., Gao S., Wu Y. (2021). Identification of TYR, TYRP1, DCT and LARP7 as related biomarkers and immune infiltration characteristics of vitiligo via comprehensive strategies. Bioengineered.

[36] Lay A.C., Hale L.J., Stowell-Connolly H., Pope R.J.P., Nair V., Ju W., Marquez E., Rollason R., Hurcombe J.A., Hayes B. (2021). IGFBP-1 expression is reduced in human type 2 diabetic glomeruli and modulates β1-integrin/FAK signalling in human podocytes. Diabetologia.

[37] Tervaert T.W., Mooyaart A.L., Amann K., Cohen A.H., Cook H.T., Drachenberg C.B., Ferrario F., Fogo A.B., Haas M., de Heer E. (2010). Pathologic classification of diabetic nephropathy. J. Am. Soc. Nephrol..

[38] Canney M., Gunning H.M., Zheng Y., Rose C., Jauhal A., Hur S.A., Sahota A., Reich H.N., Barbour S.J. (2022). The Risk of Cardiovascular Events in Individuals With Primary Glomerular Diseases. Am. J. Kidney Dis..

