# Supplementary material for: Immune Infiltration and Mitochondrial Function in Diabetic Kidney Disease: WGCNA and Machine Learning Identified Hub Genes with Clinical Validation
Source: Int J Mol Sci. 2026 May 23;27(11):4696. doi: 10.3390/ijms27114696 (PMC13256419; doi:10.3390/ijms27114696)
Supplement: Supplementary file 1 [file ijms-27-04696-s001.zip › ijms-4312574-supplementary.pdf]

Supplemental Online Content

Figure S1. Integration of GSE30122 and GSE104954 datasets for validation.

Table S1. Clinical characteristics

This supplemental material has been provided by the authors to give readers additional information about their work.

Figure S1. Integration of GSE30122 and GSE104954 datasets for validation.

(A) Distribution of individual datasets prior to batch correction.

(B) Distribution of the merged validation set after batch effect removal.

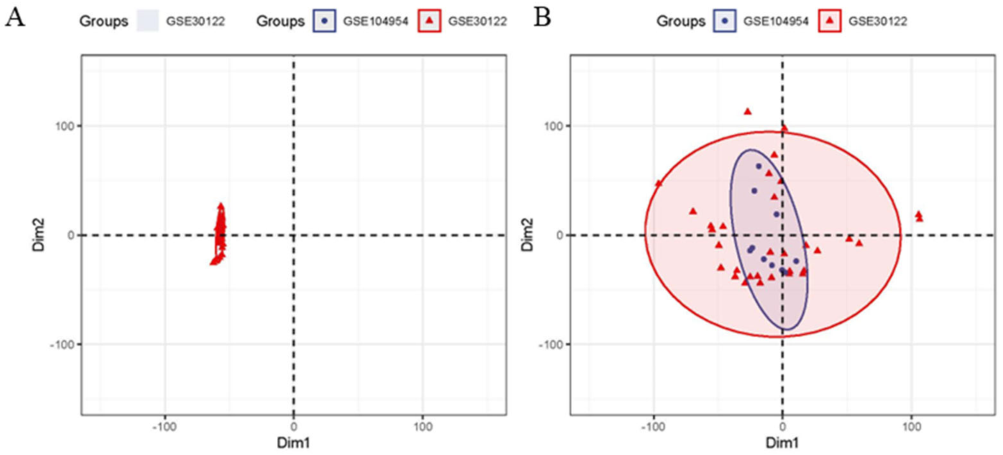

Table S1. Clinical characteristics

	Total Population (n = 70)	Low Expression (n = 23)	Medium Expression (n = 24)	High Expression (n = 23)	P
HDAC6	90.88 (66.44, 119.15)	58.28 (45.29, 65.48)	90.88 (84.51, 96.16)	126.12 (119.28, 140.67)	**<.001**
Age (years)	53.50 (45.25, 60.00)	54.00 (45.00, 58.50)	51.00 (45.75, 60.50)	53.00 (47.50, 61.50)	0.944
Male, n (%)	50 (71.429)	16 (69.565)	17 (70.833)	17 (73.913)	0.945
BMI (kg/m2)	25.452 ± 3.034	24.568 ± 3.043	25.293 ± 2.852	26.501 ± 3.018	0.091
SBP (mmHg)	145.50 (134.25, 162.25)	145.00 (131.50, 157.00)	152.50 (138.25, 173.75)	144.00 (139.00, 162.00)	0.493
DBP (mmHg)	88.00 (78.25, 95.00)	88.00 (82.00, 94.50)	87.50 (74.50, 96.25)	89.00 (80.00, 92.50)	0.976
Duration of DM (years)	10.00 (6.00, 15.00)	11.00 (6.00, 18.50)	10.00 (6.00, 15.75)	10.00 (5.50, 13.00)	0.723
Follow-up (months)	32.00 (14.00, 40.75)	22.00 (11.00, 40.00)	37.50 (13.00, 52.00)	30.00 (16.50, 37.50)	0.581

Renal Composite Endpoint, n (%)	40 (57.143)	9 (39.130)	14 (58.333)	17 (73.913)	**0.048**
Major Cardiorenal Event, n (%)	53 (75.714)	13 (56.522)	19 (79.167)	21 (91.304)	**0.02**
Scr (μmol/L)	94.8(90.3, 159.71)	102.40 (71.85, 140.95)	100.95 (81.60, 160.78)	134.00 (108.10, 201.60)	**0.035**
eGFR (ml/min/1.73m²)	57.36 (35.80, 86.39)	75.46 (54.32, 98.04)	64.10 (38.81, 92.73)	37.00 (29.62, 62.50)	**0.014**
BUN (mmol/L)	8.84 (7.21, 10.46)	7.99 (5.99, 10.21)	8.52 (7.17, 11.87)	9.53 (8.30, 13.48)	**0.049**
Uric acid (mmol/L)	377.50 (319.75, 426.25)	372.00 (315.00, 425.50)	372.00 (317.75, 417.50)	404.00 (353.00, 436.50)	0.623
ALB (g/L)	30.70 (23.88, 35.58)	28.30 (22.85, 35.05)	29.25 (24.25, 34.17)	31.70 (27.15, 36.05)	0.511
HbA1c (%)	7.50 (6.60, 8.80)	7.09 (6.40, 8.05)	7.50 (6.40, 8.83)	7.93 (7.10, 9.10)	0.180
TC (mmol/L)	5.52 (4.46, 6.70)	5.77 (4.74, 6.80)	5.10 (4.24, 5.62)	6.06 (4.96, 7.29)	0.087
TG (mmol/L)	1.55 (1.19, 2.30)	1.46 (1.10, 2.31)	1.44 (1.16, 1.74)	1.94 (1.48, 3.33)	0.088
HDL-C (mmol/L)	1.14 (0.93, 1.47)	1.09 (0.87, 1.50)	1.09 (0.99, 1.23)	1.21 (0.95, 1.58)	0.562
LDL-C (mmol/L)	3.49 ± 1.30	3.08 ± 1.24	3.40 ± 1.07	4.01 ± 1.44	**0.044**
24hUP (g/24h)	2.86 (1.26, 7.61)	2.81 (1.42, 9.96)	2.64 (1.43, 5.55)	4.40 (1.12, 7.17)	0.616
RBP (mg/L)	54.75 (45.65, 70.75)	48.90 (37.05, 76.25)	49.85 (46.13, 66.12)	64.00 (53.85, 76.05)	0.130
ALP (U/L)	73.75 (63.20, 90.00)	74.00 (65.00, 90.85)	64.35 (56.75, 79.75)	84.00 (69.50, 100.85)	**0.039**
Hb (g/L)	111.00 (99.00, 128.00)	113.00 (101.00, 136.00)	107.50 (98.50, 121.25)	111.00 (99.00, 137.50)	0.573
Urine specific gravity	1.01 (1.01, 1.02)	1.01 (1.01, 1.02)	1.01 (1.01, 1.02)	1.01 (1.01, 1.01)	0.173

Calcium (mmol/L)	2.12 (2.00, 2.24)	2.09 (1.94, 2.24)	2.08 (1.98, 2.25)	2.16 (2.05, 2.21)	0.722
25(OH)D (nmol/L)	22.20 (13.93, 35.20)	17.60 (11.50, 28.35)	27.45 (16.80, 45.30)	18.90 (12.80, 33.10)	0.103
Phosphorus (mmol/L)	1.267 ± 0.273	1.294 ± 0.356	1.293 ± 0.204	1.213 ± 0.244	0.52
PTH (pg/ml)	46.65 (26.98, 65.88)	36.60 (30.85, 55.30)	52.15 (32.17, 56.80)	53.70 (24.20, 76.65)	0.608
IgA (g/L)	2.36 (1.75, 3.08)	2.34 (1.67, 3.13)	2.28 (1.98, 3.01)	2.47 (1.89, 3.01)	0.979
IgG (g/L)	8.74 (7.14, 11.67)	8.22 (6.03, 10.95)	9.46 (7.52, 11.85)	8.75 (7.67, 12.25)	0.275
Complement C3 (g/L)	0.98 ± 0.22	0.86 ± 0.20	1.03 ± 0.19	1.06 ± 0.21	**0.003**
Complement C4 (g/L)	0.28 (0.23, 0.32)	0.28 (0.25, 0.33)	0.28 (0.24, 0.30)	0.28 (0.21, 0.32)	0.756
Cystatin C (mg/L)	1.73 (1.18, 2.00)	1.28 (1.06, 1.79)	1.48 (1.17, 1.98)	1.76 (1.73, 2.32)	**0.025**
Urine NAG (U/L)	15.20 (10.50, 22.98)	14.00 (10.20, 23.45)	16.70 (9.80, 23.35)	15.10 (11.85, 22.60)	0.910
Serum NGAL (ng/ml)	159.68 (117.00, 168.17)	159.68 (76.45, 159.68)	159.68 (114.05, 233.93)	159.68 (157.84, 159.68)	0.300
Urine NGAL (ng/ml)	60.57 (25.00, 60.57)	60.57 (38.00, 60.57)	59.84 (25.00, 60.57)	60.57 (60.57, 60.57)	0.283
Urine pH (<5 / 5-8 / >8)	18/40/12	2/18/3	6/15/3	2010/7/6	**0.016**
Urine bicarbonate (mmol/L)	25.00 (24.25, 27.00)	25.86 (24.00, 27.00)	25.86 (25.00, 27.00)	25.00 (24.00, 26.00)	0.532
Urine titratable acid (mmol/L)	9.00 (5.25, 14.75)	8.00 (5.50, 15.00)	10.00 (6.75, 12.00)	9.00 (5.00, 13.50)	0.893
Urine ammonium (mmol/L)	16.50 (12.00, 20.53)	18.00 (13.00, 22.00)	16.50 (12.00, 19.58)	16.00 (9.00, 17.00)	0.181
Pathological Stage (I/II/III/IV)	1/21/36/12	0/11/11/1	1/6/14/3	0/4/11/8	**0.043**
IFTA (0/1/2/3)	2/30/14/24	1/12/5/5	0/11/3/10	1/7/9/9	0.456
IF (0/1/2)	0/50/20	0/15/3	0/15/9	0/20/8	**0.013**
Vascular Lesion (0/1/2)	16/16/38	2006/8/9	2006/5/13	2004/3/16	0.302

Glomerulosclerosis (%)	30.854 ± 27.092	30.587 ± 28.092	20.268 ± 21.354	42.166 ± 27.931	**0.019**
RAAS inhibitors, n (%)	55 (78.571)	17 (73.913)	20 (83.333)	18 (78.261)	0.717
Glucose-lowering drugs, n (%)	47 (67.143)	13 (56.522)	15 (62.500)	19 (82.609)	0.142
Insulin, n (%)	53 (75.714)	18 (78.261)	19 (79.167)	16 (69.565)	0.701
Calcium channel blockers, n (%)	53 (75.714)	20 (86.957)	18 (75.000)	15 (65.217)	0.227
Beta-blockers, n (%)	15 (21.429)	5 (21.739)	4 (16.667)	6 (26.087)	0.717
Diuretics, n (%)	6 (8.571)	4 (17.391)	2 (8.333)	0 (0.00)	0.113
Lipid-lowering drugs, n (%)	33 (47.143)	12 (52.174)	8 (33.333)	13 (56.522)	**0.237**

Data are presented as mean ± SD, median (IQR), or n (%). P-values in bold indicate statistical significance ($P < 0.05$). Abbreviations: BMI, body mass index; SBP, systolic blood pressure; DBP, diastolic blood pressure; DM, diabetes mellitus; Scr, serum creatinine; eGFR, estimated glomerular filtration rate; BUN, blood urea nitrogen; ALB, albumin; HbA1c, hemoglobin A1c; TC, total cholesterol; TG, triglyceride; HDL-C, high-density lipoprotein cholesterol; LDL-C, low-density lipoprotein cholesterol; 24hUP, 24-hour urine protein; RBP, retinol binding protein; ALP, alkaline phosphatase; Hb, hemoglobin; 25(OH)D, 25-hydroxyvitamin D; PTH, parathyroid hormone; NAG, N-acetyl-β-D-glucosaminidase; NGAL, neutrophil gelatinase-associated lipocalin; IFTA, interstitial fibrosis and tubular atrophy; IF, interstitial inflammation.
